# Effect of High Pressure on the Reducibility and Dispersion of the Active Phase of Fischer–Tropsch Catalysts

**DOI:** 10.3390/ma12121915

**Published:** 2019-06-13

**Authors:** Simón Yunes, Miguel Ángel Vicente, Sophia A. Korili, Antonio Gil

**Affiliations:** 1 Micromeritics Instrument Corporation, 4356 Communications Drive, Norcross, GA 30093, USA; Simon.Yunes@micromeritics.com; 2 GIR QUESCAT-Departamento de Química Inorgánica, Universidad de Salamanca, 37008 Salamanca, Spain; mavicente@usal.es; 3 INAMAT-Departamento de Ciencias, Universidad Pública de Navarra, 31006 Pamplona, Spain; sofia.korili@unavarra.es

**Keywords:** Fischer–Tropsch, supported iron oxide, supported cobalt oxide, reducibility, dispersion

## Abstract

The effect of high pressure on the reducibility and dispersion of oxides of Co and Fe supported on γ-Al_2_O_3_, SiO_2_, and TiO_2_ has been studied. The catalysts, having a nominal metal content of 10 wt.%, were prepared by incipient wetness impregnation of previously calcined supports. After drying at 60 °C for 6 h and calcination at 500 °C for 4 h, the catalysts were reduced by hydrogen at two pressures, 1 and 25 bar. The metal reduction was studied by temperature-programmed reduction up to 750 °C at the two pressures, and the metal dispersion was measured by CO chemisorption at 25 °C, obtaining values between 1% and 8%. The physicochemical characterization of these materials was completed by means of chemical analysis, X-ray diffraction, N_2_ adsorption-desorption at −196 °C and scanning electron microscopy. The high pressure lowered the reduction temperature of the metal oxides, improving their reducibility and dispersion. The metal reducibility increased from 42%, in the case of Fe/Al_2_O_3_ (1 bar), to 100%, in the case of Fe/TiO_2_ (25 bar).

## 1. Introduction

Supported cobalt and iron catalysts have been extensively studied in the Fischer–Tropsch reaction for the conversion of synthesis gas obtained from natural gas due to their high activity, high selectivity to long-chain paraffins, and low activity in the formation of water [1]. Despite this huge amount of research work, there is still no agreement in the scientific community about the active phases in the reaction between CO and H_2_. Thus, for example, in the case of Fe catalysts there is a high degree of consensus about the fact that its carbides, and not metallic Fe, are the active phase. Likewise, the two factors that can control the activity of the catalysts are the degree of reduction of the metallic precursor as well as the shape and size of the metal particles formed, characteristics that are related through the dispersion, the distribution of the particles on the support [2].

In general, the type and structure of the support affect the dispersion, particle size, and reducibility, and as a consequence the activity of the supported metal catalysts [2,3]. The acidity of the supports, as well as the presence of dopants, are other factors that affect the reducibility and dispersion of the metallic phase [2,4]. Other influencing preparation variables are the metal precursor, the solvent, the metal content, the method of preparation, and the pretreatments before the catalytic tests. For example, in the case of cobalt, CoO can react with the supports both during the synthesis and during the reduction treatment, resulting in various mixed compounds like CoAl_2_O_4_, Co_2_SiO_4_ or CoTiO_3_ [5,6,7,8,9,10]. These mixed compounds require far too high reduction temperatures to reduce the metal. Under this way, cobalt metal can be lost by sublimation and increase the particle sizes by sintering, resulting in catalysts with lower performance. The reduction temperatures can be decreased by increasing the pressure, also allowing less cobalt to be lost, and decreasing sintering. Other possible action is the promotion with noble metals (Pd, Pt, Re, and Ru have been used) to enhance the reducibility of the oxides, to improve the metal dispersion, to reduce the catalyst deactivation, etc. [11].

The reaction conditions, such as temperatures up to 350 °C, pressures up to 55 bar, and the presence of fluids under supercritical conditions, also control the Fischer–Tropsch synthesis process [12]. It is highly recommended to characterize the catalysts under these conditions to have a more realistic view of the effect of the different variables on the properties of the solids.

The aim of this work is to evaluate the effect of pressure on the reducibility and dispersion of Co and Fe catalysts prepared from various supports. Some examples of this effect have been published previously but only for cobalt catalysts supported on carbon nanofibers [13,14]. In this work, a comparative study also considering Fe as active phase and other catalytic supports is presented.

## 2. Materials and Methods

### 2.1. Preparation of the Catalysts

The Co and Fe catalysts, with a nominal metal content of 10 wt.%, were prepared by incipient wetness impregnation of the supports. The salts used were Co(NO_3_)_2_•6H_2_O (Panreac, Castellar del Vallés, Barcelona, Spain) and Fe(NO_3_)_3_•9H_2_O (Riedel-de Haën-Honeywell, Madrid, Spain), respectively. The commercial supports used were γ-Al_2_O_3_ (Spheralite 505, Procatalyse, Rueil Malmaison, France), SiO_2_ (Aerolyst 350, Degussa, Frankfurt, Germany), and TiO_2_ (Aeroxide TiO_2_ P25, Degussa). Prior to their use, all of them were calcined in air at 500 °C for 4h. After impregnation, all the catalysts were dried at 60 °C for 6 h and calcined again at 500 °C for 4 h.

### 2.2. Characterization Techniques

The metal content was determined by inductively coupled plasma-atomic emission spectroscopy (ICP-AES) using Varian Vista-MPX equipment with radial vision (Varian, Palo Alto, CA, USA). The crystalline structure of the catalysts was analyzed by X-ray diffraction (XRD) in a Siemens D5000 diffractometer (Siemens, Munich, Germany). The scanning electron microscopy analyses were carried out at CLPU (Salamanca, Spain) using a Carl Zeiss SEM EVO HD25 (Zeiss Microscopy, Jena, Germany). The textural characterization of the supports and the catalysts synthesized was carried out by adsorption-desorption of N_2_ (Air Liquide, 99.999%) at −196 °C using a static volumetric method in Micromeritics model ASAP 2010 equipment (Norcross, GA, USA).

**Figure 1:**
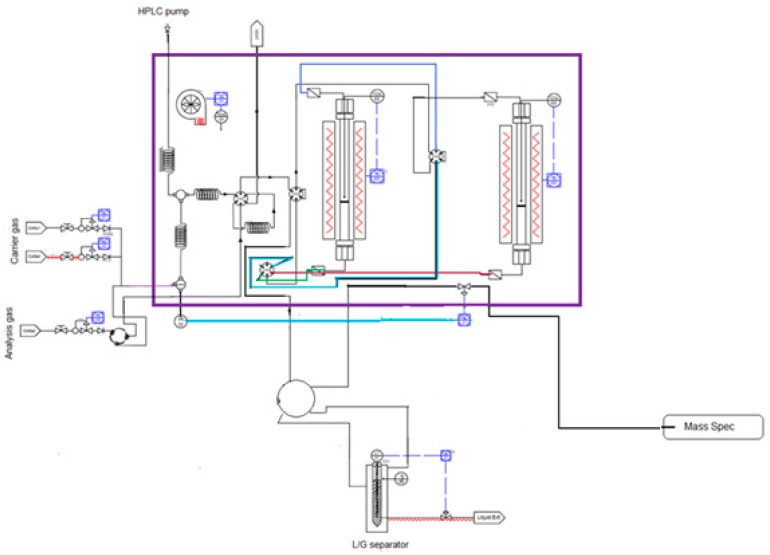
Flow-diagram of the Micro Catalyst Characterization and Testing Center (MCCTC) connected to a mass spectrometer.

The temperature-programmed reduction (TPR) and the pulse chemisorption experiments were carried out in an automated and controlled Effi Microactivity-reference PID Eng & Tech, denoted as Micro Catalyst Characterization and Testing Center, MCCTC, Madrid, Spain (see Figure 1). This equipment is mainly used to carry out catalytic reactions of any kind at a pressure between atmospheric and 200 bar. The configuration of the system allows to characterize the catalyst in situ, especially when carrying out catalytic tests in which the deactivation of the catalyst takes place. Under these conditions, it is necessary to characterize the catalyst without removing it from the reactor, avoiding its contact with the atmosphere.

The equipment consists of a hot box where all the pipes, valves, furnace, and reactor are arranged, normally kept warm at a temperature up to 200 °C to prevent the condensation of vapors. The equipment is connected in line to an MKS Instruments mass spectrometer, CirrusTM single-quadrupole model, which constitutes the system’s gas analysis system (Andover, MA, USA).

The system has a tubular steel reactor of 32 mm in length and 9 mm in internal diameter, with the catalytic bed placed inside it on a porous plate. The catalytic bed is fixed between two layers of quartz wool. The reactor is located inside a longitudinal opening cylindrical furnace that allows the thermal regulation of the system at a maximum working temperature of 1100 °C. A type K thermocouple placed in contact with the catalytic bed, which is part of the temperature measurement and control system, is placed inside the reactor. The flow of gases enters the reactor in a descending way and the reaction products exit from the bottom, being then introduced into the analysis system. The flow rate of all the gases entering the reaction system is measured through mass flow controllers. The gases are preheated in the hot box of the system and directed towards a six-way valve that pneumatically directs the path of the current directly to the reactor tube or to the gas outlet. In this valve, a loop (0.521 cm^3^ NTP) was adapted, which allows to dose an active gas such as CO in order to determine the dispersion of the active phase of any type of catalyst.

For the TPR experiments, 0.3 g of catalyst was pretreated by heating from room temperature to 150 °C at a heating rate of 10 °C/min, and maintained at 150 °C for 2 h, all the process under a flow of N_2_ (99.999%) of 50 cm^3^/min. The samples were then reduced using a 2.5% H_2_/Ar (99.999%) mixture with a flow of 400 cm^3^/min, from room temperature to 750 °C, with a heating rate of 10 °C/min, and a pressure of 1 and 25 bar. This temperature was maintained until the baseline returned to zero, indicating a complete reduction. Two commercial pure oxides, Co_3_O_4_ (Sigma-Aldrich España, Madrid, Spain, 99%) and Fe_2_O_3_ (Sigma-Aldrich, 99.99%), were used as references for calibrating the reduction profiles and quantifying the amount of H_2_ consumed by the catalysts studied.

The dispersion of the active phase was determined by repeating the reduction treatment, both at 1 and 25 bar pressure, but finishing it at 500 °C and maintaining this temperature for 4 h to ensure that the baseline was recovered. Next, the reducing mixture was replaced by an inert gas at the same temperature to ensure the elimination of all traces of H_2_. In the next step, the temperature was lowered to room temperature, and the dosing was carried out using a calibrated loop of 0.521 cm^3^ of CO (99.999%) until the saturation of the signal was observed, almost three identical peaks. With the volume of chemisorbed CO, the metallic content and the stoichiometric ratio CO/metal, 1:1 in this case, the percent dispersion of the metals was determined using the following Equation (1),

(1) $$D\left(\%\right)=\;\frac{V_{ads}\cdot f_{a}\cdot W_{a}}{V_{mol}\cdot M\left(\%\right)}$$

where *D*(%) is the metal dispersion, *V_ads_* is the total volume of CO chemisorbed, *f_a_* is the stoichiometry factor, *W_a_* is the atomic mass of the active metal, *V_mol_* is the molar volume of the CO and *M*(%) is the mass percent of metal present in the catalysts.

In the case of the chemisorption temperature, the value was selected to avoid the contribution to the physical adsorption of CO on the supports. For the stoichiometric relationship, it was assumed that each molecule of CO interacted with one Co or Fe atom on the surface [15,16].

## 3. Results and Discussion

**Figure 2:**
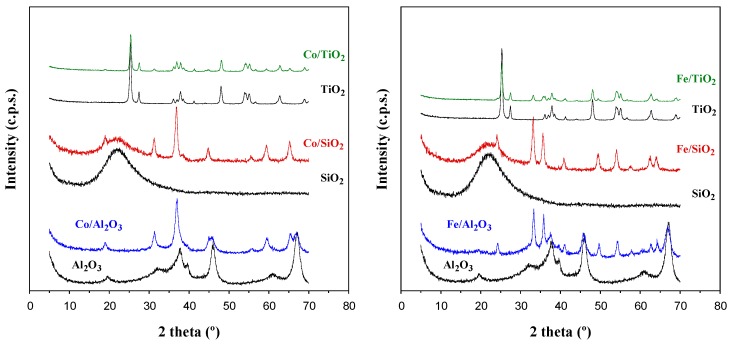
XRD patterns of the supported cobalt (**left**) and iron (**right**) oxide catalysts.

The XRD results indicated (see Figure 2) the formation of the cobalt spinel Co_3_O_4_ in the case of the cobalt catalysts and the hematite phase Fe_2_O_3_ in the case of iron catalysts. No mixed phases involving the supports and the active phases were detected, although in the case of alumina, Al(III) cations from the support should isomorphically incorporate to both active phases, and in fact reducibility studies strongly suggested this possibility (vide infra).

**Figure 3:**
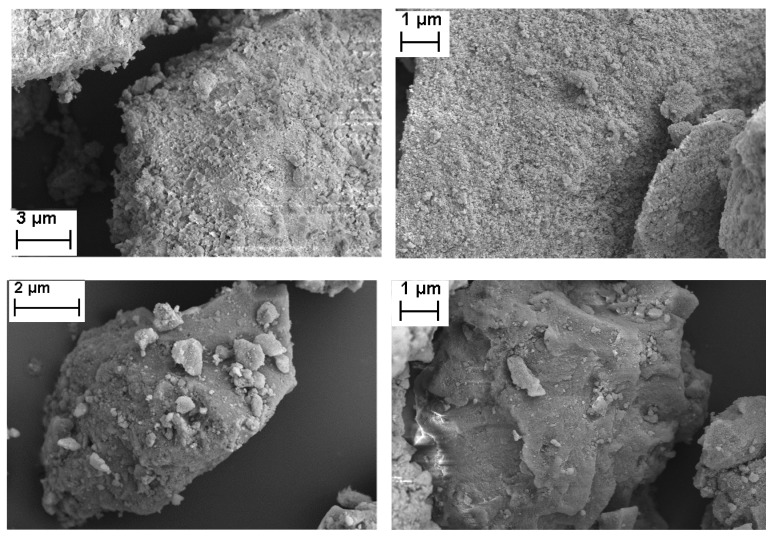

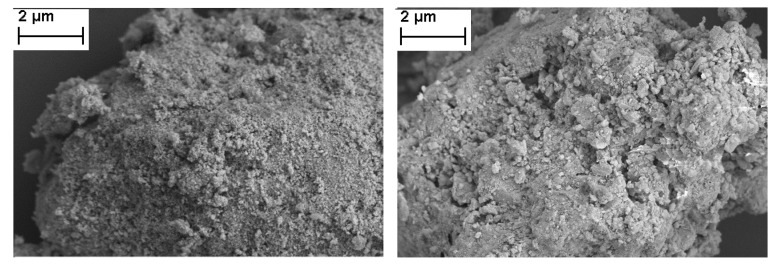
SEM images of the supported cobalt (**left**) and iron (**right**) oxide catalysts. Top: Alumina-supported catalysts; middle: Silica-supported catalysts; bottom: Titania-supported catalysts.

Selected micrographs of the cobalt catalysts are included in Figure 3. The images confirmed that the structures of the particles of the supports were not significantly modified by the impregnation with the metallic solutions and the subsequent calcination.

The adsorption isotherms were of type II according to the IUPAC classification [17] for γ-Al_2_O_3_ and TiO_2_ supports and type IV in the case of SiO_2_ support. The presence of metallic oxides did not change the shape of the adsorption isotherms or the shape of the hysteresis cycles that originated in the desorption process. This result may indicate that the metal oxides were well dispersed on the surface of the supports. The textural properties, specific surface area, and pore volume of the supports and of the catalysts are included in Table 1, in which the metal content of the catalysts is also included.

**Figure 4:**
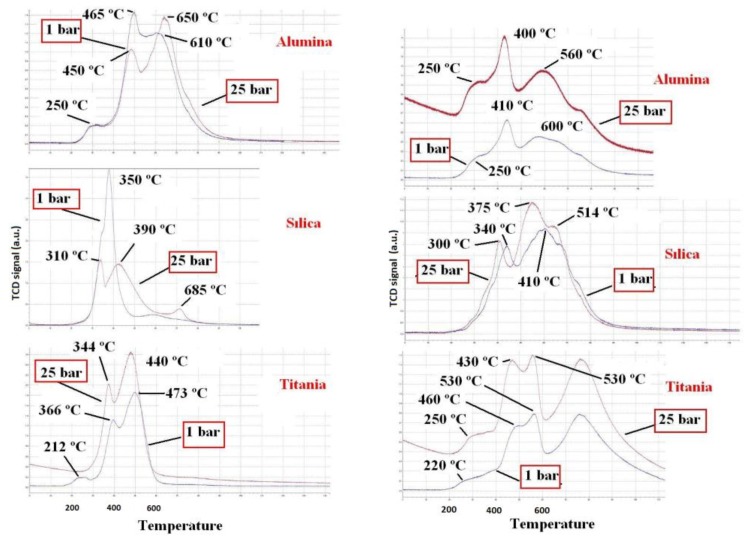
Reduction profiles corresponding to the supported cobalt (**left**) and iron (**right**) oxide catalysts.

The TPR profiles corresponding to the series of Co catalysts are included in Figure 4. In all the cases, a reduction peak can be observed at 350–465 °C, which coincided with the maximum peak of reduction of pure oxide Co_3_O_4_ to Co^0^. The reduction shoulder observed at lower temperature can be related to the reduction of Co_3_O_4_ to metallic Co in two steps (Co^3+^ → Co^2+^ → Co^0^), as has been discussed by Arnoldy and Moulijn [18]. These results confirmed the presence of Co_3_O_4_. In the case of the catalyst with Al_2_O_3_ as support, the higher reduction temperatures may be due to a greater oxide-support interaction. Al(III) can isomorphically incorporate to the spinel phase. The peak of reduction centered at higher temperature (600–1000 °C), which was observed in the Co/Al_2_O_3_ catalyst, suggested the reduction of Co^2+^ species present in the form of cobalt spinels.

In two previous works, Jacobs et al. [19] and Borg et al. [20] reported that the metal-support interactions affected the reduction of cobalt species and the strength of such interactions for various supports decreased in the order Al_2_O_3_ > TiO_2_ > SiO_2_. In the case of Al_2_O_3_ and as a result of the metal-support interactions that arose from the diffusion of cobalt ions into alumina lattice sites of octahedral or tetrahedral geometry, the formation of CoAl_2_O_4_ was proposed, and as a consequence the reducibility of the cobalt was hindered. TiO_2_ is known to exhibit the strong metal-support interaction (SMSI) effect, where during the reduction of the cobalt oxide, the partial reduction of the support also takes place, encapsulating or decorating the cobalt particles [21]. The nature of hydroxyl groups and their concentration and distribution on the silica surface play the main role in the dispersion of cobalt particles. In this type of support, the formation of Co_2_SiO_4_ has also been reported.

The TPR profiles corresponding to the series of Fe catalysts are also included in Figure 4. The reduction of Fe_2_O_3_ was related to a first peak at 400–460 °C (3Fe_2_O_3_ → 2Fe_3_O_4_) and a second broader peak located at high temperatures that reflected the reduction to metallic iron (2Fe_3_O_4_→6Fe). The shoulder at 630 °C indicated an intermediate reduction state (2Fe_3_O_4_ → 6FeO → 6Fe).

The volumes of H_2_ consumed by weight of metal oxide, the degree of reducibility of the catalysts, and the dispersion of the metal phases for the catalysts studied are included in Table 2. The results are given for the two working pressures, 1 and 25 bar.

The low values of metal dispersion can be related to the high metal contents of the synthesized catalysts. It was also indicative of the large size of the metal particles. For the two metals considered, the catalysts synthesized using TiO_2_ as support had greater degrees of oxide reduction. This result can be attributed to the weak interaction existing between the metallic oxide and the support and characteristic of the SMSI effect. The situation was very different for the catalysts supported on γ-Al_2_O_3_, which showed lower degrees of oxide reduction, behavior that can be explained through strong oxide-support interactions, even with the formation of spinels involving cations from the support, that is, Fe(II) or Co(II) as divalent cations, and Fe(III) or Co(III) from the precursors and Al(III) from the support as trivalent cations, this effect seeming to be more significant in the case of Co-samples [5,18]. These effects decreased with pressure, as the reducibility of the metal oxides and the metal dispersion increased.

## 4. Conclusions

The effects of the high pressure of on the reducibility of catalysts based on cobalt and iron oxides on three commercial supports, γ-Al_2_O_3_, SiO_2_, and TiO_2_, have been presented. Two effects have been observed, the high pressure lowered the reduction temperature, decreasing the sintering of the metal oxide particles, while the pressure improved the reducibility of the metal oxides to an almost total reduction value. These two effects gave rise to a greater dispersion of the active metal phase, which may result in an increase in the activity of the catalysts.

## Figures and Tables

**Table 1:** Textural properties derived from N_2_ adsorption at −196 °C and metal content by inductively coupled plasma-atomic emission spectroscopy ICP-AES.

Catalyst	S_BET_ (m^2^/g)	V_p_ (cm^3^/g) *	Metal Content (wt.%)
γ-Al_2_O_3_	183	0.410	--
SiO_2_	288	0.797	--
TiO_2_	51	0.147	--
Co/Al_2_O_3_	122	0.291	13.54
Co/SiO_2_	225	0.615	13.79
Co/TiO_2_	32	0.251	10.90
Fe/Al_2_O_3_	132	0.290	9.11
Fe/SiO_2_	220	0.562	13.30
Fe/TiO_2_	38	0.235	9.62

* Total pore volume, calculated from N_2_ adsorption at p/p^o^ = 0.98.

**Table 2:** Results of H_2_ consumption, metal reducibility and dispersion.

Catalyst	Q(H_2_) (cm^3^/g_oxide_)	R (%)	D (%)
Co/TiO_2_ (25 bar)	372	100	5
Co/TiO_2_ (1 bar)	315	85	1
Co/SiO_2_ (25 bar)	299	80	2
Co/SiO_2_ (1 bar)	296	80	1
Co/Al_2_O_3_ (25 bar)	296	80	6
Co/Al_2_O_3_ (1 bar)	271	73	3
Fe/TiO_2_ (25 bar)	421	100	7
Fe/TiO_2_ (1 bar)	306	73	1
Fe/SiO_2_ (25 bar)	346	82	3
Fe/SiO_2_ (1 bar)	295	70	1
Fe/Al_2_O_3_ (25 bar)	236	56	8
Fe/Al_2_O_3_ (1 bar)	175	42	3
